# Scleraxis genes are required for normal musculoskeletal development and for rib growth and mineralization in zebrafish

**DOI:** 10.1096/fj.201802654RR

**Published:** 2019-05-17

**Authors:** Erika Kague, Simon M. Hughes, Elizabeth A. Lawrence, Stephen Cross, Elizabeth Martin-Silverstone, Chrissy L. Hammond, Yaniv Hinits

**Affiliations:** * Department of Physiology, Pharmacology, and Neuroscience, Medical Sciences, University of Bristol, Bristol, United Kingdom;; † Randall Centre for Cell and Molecular Biophysics, School of Basic and Medical Biosciences, Faculty of Life Sciences and Medicine, King’s College London, London, United Kingdom

**Keywords:** zebrafish, tendon, ribs, craniofacial, muscle

## Abstract

Tendons are an essential part of the musculoskeletal system, connecting muscle and skeletal elements to enable force generation. The transcription factor scleraxis marks vertebrate tendons from early specification. *Scleraxis*-null mice are viable and have a range of tendon and bone defects in the trunk and limbs but no described cranial phenotype. We report the expression of zebrafish scleraxis orthologs: scleraxis homolog (*scx)-a* and *scxb* in cranial and intramuscular tendons and in other skeletal elements. Single mutants for either *scxa* or *scxb,* generated by clustered regularly interspaced short palindromic repeats (CRISPR)/CRISPR-associated protein 9 (Cas9), are viable and fertile as adult fish. Although *scxb* mutants show no obvious phenotype, *scxa* mutant embryos have defects in cranial tendon maturation and muscle misalignment. Mutation of both scleraxis genes results in more severe defects in cranial tendon differentiation, muscle and cartilage dysmorphogenesis and paralysis, and lethality by 2–5 wk, which indicates an essential function of scleraxis for craniofacial development. At juvenile and adult stages, ribs in *scxa* mutants fail to mineralize and/or are small and heavily fractured. *Scxa* mutants also have smaller muscle volume, abnormal swim movement, and defects in bone growth and composition. Scleraxis function is therefore essential for normal craniofacial form and function and vital for fish development.—Kague, E., Hughes, S. M., Lawrence, E. A., Cross, S., Martin-Silverstone, E., Hammond, C. L., Hinits, Y. Scleraxis genes are required for normal musculoskeletal development and for rib growth and mineralization in zebrafish.

The musculoskeletal system is formed by coordinated differentiation and morphogenesis of skeletal muscle, tendon, ligament, cartilage, bone, and associated joint cell types (reviewed in refs. 1 and 2). Recent studies have shown that both signaling between component tissues and physical forces deriving from muscular contraction or passive load can remodel various elements of the system to fit form to function (reviewed in ref. 3). In the integrated musculoskeletal system, resolving the primary cause of defects requires identification of the earliest failures—in muscle, bone, or tendon—and determination of their secondary consequences during development of the entire system. With such detailed understanding, treatments for genetic or environmentally induced musculoskeletal pathologies may be more effective.

Tendons play an essential role in muscular control of the body by connecting muscle and skeletal elements, allowing force transmission. The earliest and most persistent known marker for the tendon cell lineage is the basic helix-loop-helix (bHLH) transcription factor scleraxis (scx) (4–6). *Scx* expression marks a somitic compartment called the syndetome in mammals and birds, from which the tendon precursors are derived (7). *Scx* is also expressed in pharyngeal arches and facial tendons of mouse embryos (8–10). *Scx*-null mice are viable but show a dramatic defect in tendon differentiation, resulting in a loss of intermuscular tendons and the tendons responsible for transmitting musculoskeletal force in the limbs, tail, and trunk. However, short-range muscle-anchoring tendons, such as the ones anchoring the intercostal muscles to the ribs, and ligaments, like the cruciate ligaments of the knee, are unaffected (11). *Scx* is not required for tendon cell specification, as tendon progenitors are present in *Scx*^−/−^ mutant mice, but *Scx* is necessary for the condensation and differentiation of tendon cell populations (11). Experiments in mice and chicks have shown that *Scx* is required for secretion of structural extracellular matrix (ECM) components, including *col1a1*, other tendon-associated collagens, and tenomodulin (*Tnmd*) (12–14), for formation of extended cytoplasmic extensions that support matrix organization and in the crosstalk between tenocytes and endotenon cells (11).

*Scx* is also essential for developmental events beyond tendons themselves. Scx is required for normal development of entheses, which serve as insertion points for tendons onto bones (14–17). Most recently, scx was also implicated in regulation of fracture callus formation during bone healing (18). Thus, scx promotes several aspects of musculoskeletal development in amniotes.

It has been suggested that *Scx* also functions in tendons of lower vertebrates. In frogs, *Scx* accumulates at the end of muscle fibers in the somites and the limbs and is involved in inducing tendon matrix genes tenascin C and betaig-h3 (19, 20). In trout and zebrafish, the myosepta, sheets of connective tissue that separate the somite muscle blocks, are initially acellular but later contain cells expressing *col1a1* and *Scx* (21, 22). Zebrafish have 2 *Scx* genes, *scxa* and *scxb*, but only *scxa* expression has been described (23, 24). Zebrafish *scxa*-positive cranial tenocytes are located between muscles and the craniofacial skeleton and coexpress tendon markers such as *tnmd*, *col1a2*, and *trsp4b* (23–26). Interestingly, cranial and fin tendon progenitors can be induced in the absence of muscle or cartilage, whereas myoseptal *scxa* expression requires muscle for its initiation (23), which suggests that these tendon populations have different origins and regulation. As in mammals, zebrafish have neural crest–derived craniofacial tendons and ligaments (8, 23, 24, 26, 27). However, knockdown experiments in zebrafish with antisense morpholino oligonucleotides against both *scxa* and *scxb* were reported to have no effect on *tnmd* expression or create any craniofacial defects in the embryos (23). Although expression data implicate *Scx* in tendon development in various vertebrate groups, functional data derive mostly from mice trunk and limbs. We set out to create a zebrafish loss-of-function model to increase understanding of Scx function across vertebrates.

Here we describe the differential expression of *scxa* and *scxb* in embryonic, juvenile, and adult zebrafish. Zebrafish single mutants for *scxa* or *scxb* are each viable, allowing assessment of the adult musculoskeletal system. Mutations in *scxa* lead to embryonic defects in cranial tendon composition and shape as well as muscle misalignment. At juvenile and adult stages, *Scxa* is essential for growth and mineralization of the ribs and is required for normal swim movement, muscle volume, and body composition. Lack of *Scxa* in the mutants also results in ectopic growth of bone in neural and haemal arches while also reducing jaw bone mineral density (BMD). Lack of *Scxb* alone has no obvious phenotype but exacerbates the effect of the lack of *Scxa*, indicating partially redundant function. Double mutants show severe cranial defects but no obvious defect in somitic myotendinous junctions (MTJs). Double mutant embryos have reduced muscle growth and function and paralysis of the jaw, leading to death at early juvenile stages.

## MATERIALS AND METHODS

### Generation of mutant zebrafish lines and maintenance

Clustered regularly interspaced short palindromic repeats (CRISPR)/CRISPR-associated protein 9 (Cas9) genome editing (28) was used to target *scxa* (zv11, Chr19: 3001919-3001938, GGGGGTGGCGGACGGCTGAT) and *scxb (*zv11, Chr16: 31,396,781-31,396,801, GGCTATGGTTCCTTTAAGCT) and yielded 2 nonsense alleles for *scxa* (*scxa^kg141^*, *scxa^kg170^*) and 1 for *scxb (scxb^kg107^*), all of which led to premature stop codons upstream of the bHLH domain (Supplemental Fig. S1). *scxa^kg141^* carries a 4 base pair (bp) deletion leading to a frameshift after amino acid (aa) 64, adding a tail of 2 extra incorrect aas followed by a premature stop. *scxa^kg170^* carry a 1-bp insertion also leading to a frameshift after aa 64, adding a 51 wrong aa tail before a premature stop codon in exon 1. *scxb^kg107^* is a deletion of 12 bp combined with a 27-bp insertion, which creates an immediate UAG stop codon after 45 aa of the wild-type protein (Supplemental Fig. S1). The new mutant and transgenic lines, *Tg(actc1b:egfp)^zf13^* (29) and *TgBAC(col2a1a:mCherry)^hu5910^* (30), were maintained on an Alcian blue (AB) wild-type background. Staging and husbandry were as previously described (31).

### mRNA *in situ* hybridization and immunohistochemistry

*In situ* mRNA hybridization was performed as previously described (32) and adapted for juveniles, which were cut into 3 or 4 pieces, skinned, bleached, and then treated with 50 µg/ml proteinase K for 15 min and refixed. Probes for *scxa* and *scxb* were made by amplifying from zebrafish cDNA [72 hours postfertilization (hpf); Thermo Fisher Scientific, Waltham, MA, USA] a 1151 and 847 bp fragment, respectively, and cloning them into pGEM-Teasy vector (Promega, Madison, WI, USA). The following primers were used: 5′-CAGAAAGCCGGAGGAGTGTG-3′ and 5′-TGTGTATGCGCAGAAAAAGTGAC-3′ for *scxa* and 5′-AGCAGGACTGGTTCTTCATTCTAA 3′ and 5′-CAGTGTTGCGTTCCGTTCA-3′ for *scxb*. For *tnmd* and *xirp2a* probes, the following primers (containing T3 polymerase site) were used: 5′-TCCACCCATCTCCTCTCAGA-3′ and 5′-GGATCCATTAACCCTCACTAAAGGGAATGTGGGTAGTTGCCATGGAT-3′ for *tnmd* and 5′-CTCAGCAGAGCACGGTGGAAAAC-3′ and 5′-GGATCCATTAACCCTCACTAAAGGGAAGATGGGGCGGGTTTCAAACAT-3′ for *xirp2a*. Published probes include *Sox9a* (33) and *tnnc* (34). For immunohistochemistry, the following primary antibodies were used: sarcomeric myosin heavy chain (MyHC): A4.1025, 1:10 (35), MF20 (Developmental Studies Hybridoma Bank; DSHB), 1:10, anti–green fluorescent protein, 1:500 (rabbit, Torrey Pines or chicken, ab13970; Abcam, Cambridge, MA, USA), anti-Tsp4 (Thbs4), 1:400 (ab211143, made against N-terminal recombinant fragment within zebrafish Thbs4; Abcam), and anti-α-Actinin, 1:500 (A7811; MilliporeSigma, Burlington, MA, USA). Secondary antibodies were either horseradish peroxidase–conjugated (Vector Laboratories, Burlingame, CA, USA) or Alexa dye–conjugated (Thermo Fisher Scientific). Samples for immunohistochemistry were fixed and stained as previously described (36). Wholemount pictures were taken on an Olympus DP70 camera (Tokyo, Japan), and dissected samples were flat mounted in glycerol and photographed on a Zeiss Axiophot with Axiocam (Carl Zeiss, Oberkochen, Germany) using Openlab software (Agilent, Santa Clara, CA, USA).

### Alizarin red S and Alcian blue staining

Staining was performed as previously described (37).

### Microcomputed tomography

One-year-old fish were fixed in 4% paraformaldehyde for 72 h and dehydrated to 70% ethanol. A total of 24 fish were scanned (7 *scxa^−/−^*, 3 *scxb^−/−^*, 14 siblings) using a Nikon XT H225ST computed tomography (CT) scanner (Nikon, Tokyo, Japan) at a voxel size of 21 μm (scan settings 130 kV, 150 μA, 0.5-s exposure, 3141 projections), and selected regions rescanned at 5 μm (130 kV, 53 µA, 0.7-s exposure, 3141 projections) without additional filters. Images were reconstructed using CT Pro 3D software (Nikon). Amira 6.0 (Thermo Fisher Scientific) was used to generate 3-dimensional volume renders. Soft tissues were discriminated by treating fixed fish with 2.5% phosphomolybdenic acid for 14 d, as previously described (38), followed by microCT (µCT) scanning. Muscle volume was calculated for a single transverse slice in 2 positions in the trunk: the first at the level of the midpoint of the second-to-last rib-bearing vertebra, and the second at the midpoint of the fourth vertebra posterior to the previous position. Trunk musculature was segmented and volume calculated using the Material Statistics module. Nonmuscle volume in that position was calculated by subtracting the muscle volume from the total volume.

Vertebral centrum volumes were calculated for the seventh and eighth thoracic vertebrae using the CT scans of unenhanced fish in Avizo 9.3 (Fei, Hillboro, OR, USA) by segmenting the minimum volume possible around the neural canal, excluding all processes, trabeculae, spines, and ribs in a transverse view. Volume was calculated using the Material Statistics module. The entire vertebral volume with ribs was measured on the third thoracic vertebra using the aforementioned method but by segmenting the entire vertebra, including all processes, trabeculae, spines, and ribs. BMD was quantified as previously described (39).

### Second harmonic generation imaging

Second harmonic generation (SHG) images were acquired using 10 × 0.3 numerical aperture water dipping lens, 880-nm laser excitation, and simultaneous forward and backward detection (440/20) in Leica SP8 AOBS confocal laser scanning microscope attached to a Leica DM6000 upright epifluorescence microscope with multiphoton lasers and confocal lasers allowing fluorescent and SHG acquisition of the same sample and *Z* stack. Microscope parameters for SHG acquisition were set as previously described (40). LASX (Leica Microsystems, Buffalo Grove, IL, USA) was used for image acquisition.

### Histology

Following µCT, fish were rehydrated to PBS with Tween 20 (1X PBS/0.01% Tween 20), decalcified in 1 M EDTA-solution for 20 d, embedded in paraffin, and sectioned at 8 μm. Sections were stained for AB and hematoxylin and eosin (H&E), as previously described (41). Pictures were taken using a GXML3200B with GXCAM camera (GX Vision, Stansfield, Suffolk, United Kingdom).

### Body mass index and standard weight calculation of adult fish

Adult fish from heterozygote incrosses, grown in tanks together, were anesthetized with tricaine, blotted dry, and weighed nose-to-base of tail fin, length measured with a ruler and fin-clipped for genotyping. Body mass index (BMI) was calculated as weight (g) × length^−2^ (cm). Standard weight (*K*) was calculated using Fulton’s formula: *K* = weight (g) × 100 × length^−3^ (cm) (reviewed in ref. 42).

### Fish tracking

Two or 3 fish per movie were recorded in 8 L tanks with a Nikon D3200 camera mounted above the tank at 1920 × 1080 resolution, at 24.96 frames/second (see Supplemental Video and Supplemental Fig. S7). Fish motion was quantified using the Modular Image Analysis plugin (v.0.5.17) (43) for Fiji (44, 45). Initially, the Fiji Color Deconvolution plugin (46) was used to convert the RGB-format video frames into grayscale while also enhancing the contrast of the fish from the background image of the tank. Next, the median time-projection image was subtracted from all frames to enhance the image. This image was subsequently binarized using the intermodes threshold (47) and median-filtered. Identified objects were size-filtered to remove noise spuriously detected as a fish. Individual fish were then tracked between frames using the Apache HBase implementation (Apache Software Foundation, Forest Hill, MD, USA) of the Munkres algorithm, linking costs based on centroid separation (48). From these tracks, instantaneous (frame-to-frame) speeds were calculated. To remove false tracks, tracks lasting less than 50 frames were excluded from further analysis. To measure fish curvature, the binarized objects were skeletonized and a spline curve fitted to this backbone using the Apache Math3 library (49); this permitted measurement of local curvature and backbone length.

The 100 frames in which the fish were most active were selected, and the instantaneous velocity and range of movement was compared between 6 homozygous and 6 heterozygous fish. Range of movement was calculated by subtracting the minimum curvature from the maximum curvature for each fish. Each point on the graphs corresponds to the average value for each fish, and a 2-way ANOVA was performed in GraphPad Prism v.7.04 (GraphPad Software, La Jolla, CA, USA) to compare the mean value from each heterozygous fish with the mean value from each homozygous fish.

The software is available and free to download (Supplemental File S8).

### Statistical analysis

Statistical analyses were performed using GraphPad Prism 7.00 (GraphPad Software). The tests used *n* numbers, sample sizes are indicated in the figure legends, and significant *P* values are shown on the figures. All tests met standard assumptions, and the variation between each group is shown. Sample sizes were chosen based on previous, similar experimental outcomes and were based on standard assumptions. No samples were excluded. Randomization and blinding were not used except where the genotype of zebrafish was determined after experimentation.

### Genomic and protein comparison

Clustal alignment and sequence pair distances were made using the Lasergene Genomics Suite (DNAStar, Madison, WI, USA). Analysis of synteny was made using Ensembl zebrafish zv10 and the Genomicus Synteny software v.93.01 (50)

## RESULTS

### Expression of *scxa* and *scxb* during zebrafish development

**Figure 1:**
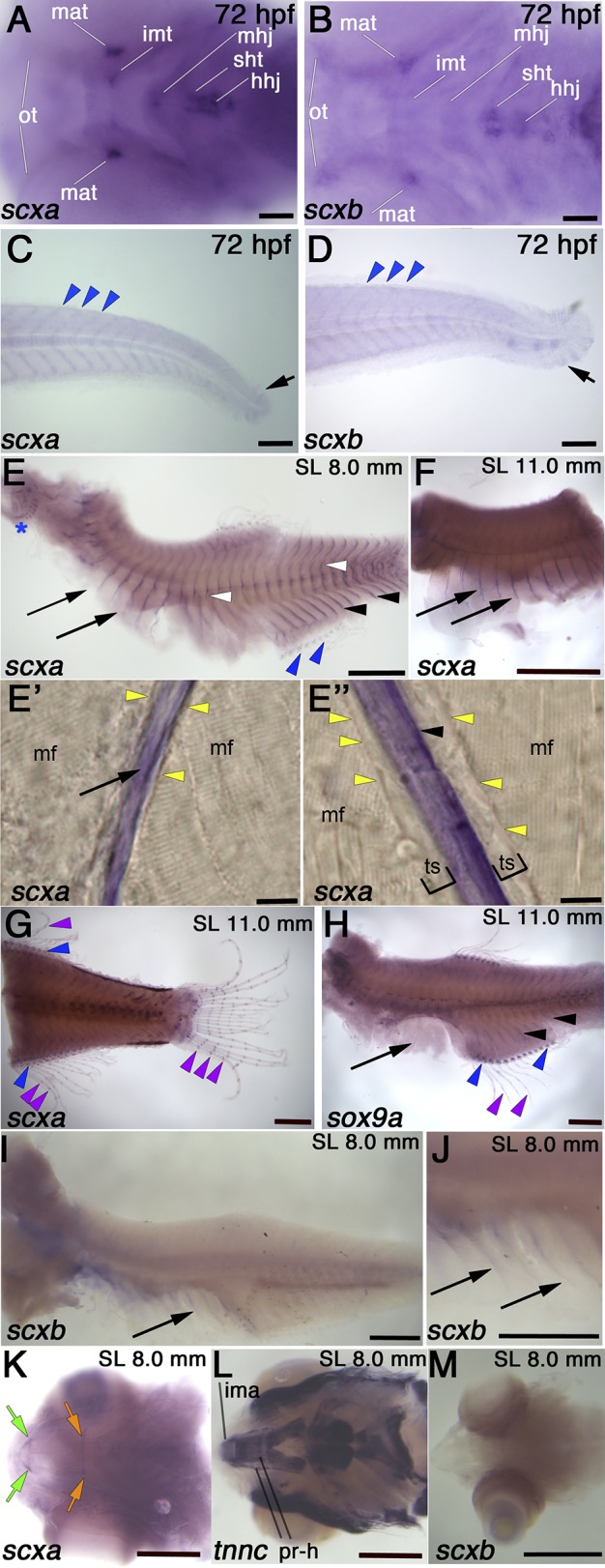
Comparison of *scxa* and *scxb* expression during embryonic and juvenile stages. *In situ* hybridization for indicated genes shown in lateral (*C*–*J*) or ventral view (*A*, *B*, *K*–*M*). *A*, *B*) At 72 hpf, *scxa* is detected in Meckel’s adductor tendon (mat), the intermandibularis tendon (imt), the mandibulohyoid junction (mhj), the sternohyoideus tendon (sht), and hyohyoideus junction (hhj). *scxb* is expressed at the sht and hhj, the mat, and the ocular muscle tendons (ots). *C*, *D*) At 72 hpf, *scxa* and *scxb* are expressed weakly at the myosepta (blue arrowheads) and the caudal fin (black arrows). *E*–*J*) *In situ* hybridization for *scxa* (*E*–*G*), *sox9a* (*H*), and *scxb* (*I*, *J*) at juvenile stages as indicated, showing expression in the trunk in the posterior vertical myosepta (arrowheads), and the more anterior myosepta near the thoracic ribs (arrows), intermuscular bones (white arrowheads), fin radials (blue arrowheads), in gills (blue asterisk), and between fin bony ray segments (purple arrowheads). Sagittal sections in rib area (*E*′) and myosepta in anal fin area (*E*″) show *scxa* staining in intramuscular tendons separated from muscle fiber (mf) ends (yellow arrowheads) by an unstained tendon-sheath or ECM (brackets). *K*–*M*) *scxa* expression at juvenile stages in head tendons, including protractor hyoideus tendons (orange arrow) and intermandibularis anterior tendons (green arrows), at the attachments of the cranial muscles (labeled with *tnnc*) (*L*): Protractor hyoideus-dorsal and ventral (pr-h) and the intermandibularis anterior (ima). No *scxb* expression is detected in cranial tendons at juvenile stages. Scale bars, 100 µm (*A*–*D*, *E*′, *E*″), 0.5 mm (*E*–*M*).

Zebrafish have 2 orthologs of the mammalian *Scx* gene, *scxa* and *scxb* (23), with Scxa and Scxb showing 68.1 and 63.6% aa identity with mouse Scx and 68.3 and 63.2% with human SCX, respectively (Supplemental Fig. S2*A*, *B* and ref. 23). Zebrafish *scxa* is syntenic to mammals, with the whole *scxa* gene positioned in the + strand inside intron 3 of *bop1* (Supplemental Fig. S2*C*, Ensembl GRCz11). In contrast, the *scxb* locus shows more rearrangements (Supplemental Fig. S2*C*, Ensembl GRCz11). We sought to know where scleraxis genes are expressed in zebrafish and whether this relates to synteny. Expression of *scxa*, but not *scxb*, has been reported (23, 24, 26, 51). By 72 hpf, both *scxa* and *scxb* mRNAs were detected in tenocytes at the junctions between skeletal elements and head muscles (**Fig. 1*A, B***). *scxb* mRNA overlapped *scxa* mRNA in the attachments of the Meckel adductor, the sternohyoideus, and ocular muscles (Fig. 1*A, B*), similar to expression of *tnmd* and *col1a2* (23). However, other tendons, such as the mandibulohyoid junction and the intermandibularis tendon, showed strong *scxa* and weak or no *scxb* expression (Fig. 1*A, B*). Strong expression of *scxb* and weak *scxa* was evident in the ocular muscle attachments (Fig. 1*A, B*). Both *scxa* and, to a lesser extent, *scxb* mRNAs are expressed at 72 hpf and beyond in somitic vertical myosepta (Fig. 1*C, D* and unpublished data). Thus, *scxa* and *scxb* expression partially overlaps in cranial tendons and ligaments and intersomitic MTJs, although *scxa* mRNA appears more abundant in both during early larval stages.

During juvenile stages, s*cxa* mRNA was detected in various skeletal elements, such as the intermuscular tendons at the vertical myosepta, the fin radials, the joints in the fin bony rays (lepidotrichia) segments at standard length (SL) 8.0–12.0 (Fig. 1*E–G*). Sections taken from stained juveniles show that *scxa* is not expressed at muscle ends at this developmental stage. Intriguingly, the chondrogenic marker *sox9a* mRNA is detected weakly in ribs, although it was observed strongly in fin endoskeletal elements (radials) and weakly in exoskeletal elements (fin rays) (Fig. 1*H*). *scxb* was only weakly detected in vertical myosepta (Fig. 1*I, J*). The head tendons and ligaments expressed strong *scxa* but little *scxb* at muscle attachments of the protractor hyoideus and intermandibularis anterior muscles that expressed the muscle marker *tnnc* (Fig. 1*K*–*M*). Thus, expression data show that *scxa* is the predominant Scx gene expressed at embryonic and juvenile stages.

### Lack of Scxa results in defective cranial tendons and ligaments and abnormal musculature

**Figure 2:**
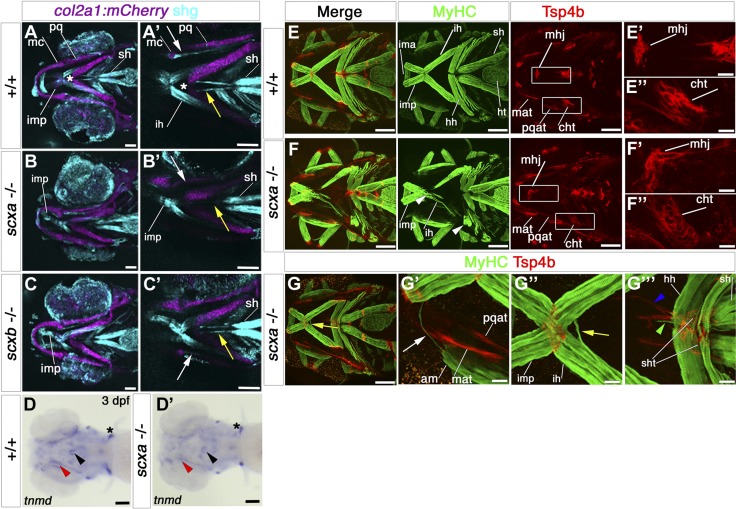
Cranial tendons, ligaments, and muscles of *scxa^−/−^* mutants are abnormal and disorganized. *A*–*C*) SHG imaging shows both collagen arrangement in tendons and ligaments and myosin heads in muscle (light blue*)* combined with confocal imaging of the transgene *TgBAC(col2a1a:mCherry)hu5910* (cartilage, purple) of 7 dpf *scxa^−/−^, scxb^−/−^*, and wild-type (+/+) larvae. Shown are representative maximum projection images of the stacks (*A*–*C*) and single scans at comparable *Z* positions (*A*′–*C*′). Decreased SHG signal was observed in the ligaments connecting the Meckel’s and palatoquadrate cartilages of *scxa* and *scxb* mutants (white arrows), and in the sternohyoideus tendon (yellow arrows) connecting the sternohyoideus muscle and the basihyal cartilage (asterisk) of *scxa* mutants. *D*) *In situ* hybridization for *tnmd* for 3 dpf *scxa^−/−^* and their siblings (+/+) showing reduced *tnmd* mRNA levels in *scxa* mutant, such as the sternohyoideus tendon (black arrowhead) and the ligaments connecting the Meckel’s and palatoquadrate cartilages (red arrowhead). Expression at the base of the cleithrum is maintained (asterisk). *E*–*G*) Confocal stacks of 4 dpf embryos from a *scxa^+/^*^−^ incross, immunostained for MyHC (A4.1025, green) and Tsp4b (red). Specific defects in tendon structure and directionality in *scxa* mutants (white rectangles) (*E*) are shown in higher magnification panels for mandibulohyoid junction (*E*′, *F*′) and ceratohyal tendon (*E*″, *F*″). *Scxa* mutants show varied array of muscle defects, such as abnormal overextension or crossing the midline of muscle fibers (*F*, *G* compared with *E*) and detached fibers in interhyoides and hyohyoides (arrowheads, *F*). The magnified area (*G*′–*G*″′) shows ectopic fiber in the adductor mandibularis (white arrow, *G*′), ectopic misguided fiber from the interhyoides is crossing the midline and growing toward the interhyoides across the midline (yellow arrow, *G*″), and ectopic extention of sternohyoides tendon (blue arrowhead) is attracting overextended fibers (green arrowhead). Am, adductor mandibularis; cht, ceratohyal tendon; hh, hyohyoides; ih, interhyoides; ima, intermandibularis anterior; imp, intermandibularis posterior; mat, Meckel’s adductor tendon; mc, Meckel’s cartilage; mhj, mandibulohyoid junction; pq, palatoquadrate cartilage; pqat, palatoquadrate adductor tendon; sh, sternohyoideus; sht, sternohyoides tendon. All images in ventral view, anterior to left. Scale bars, 100 µm, except *F*′–*F*″ and *G*′–*G*″′, 20 µm.

In contrast to the tendon and bone phenotypes that have been reported in mice lacking *Scx*, morpholino knockdown of *scxa* and *scxb* in zebrafish yielded no obvious phenotype (11, 14, 18, 23). To analyze a complete loss of function of *scxa* and *scxb* in embryonic and adult fish, we generated stable mutant lines for *scxa* and *scxb* using CRISPR/Cas9 genome editing (Supplemental Fig. S1). *Scxa^kg141^*, *scxa^kg170^*, and *scxb^kg107^* have premature stops before the bHLH domain, which is required for DNA binding and dimerization. As we have found no consistent difference between the 2 *scxa* mutant alleles, henceforth we have used the kg170 allele unless otherwise stated and refer to it as *scxa^−/−^*. For simplicity, we refer to *scxb^kg107^* mutants as *scxb^−/−^*. Analysis of the expression of *scxa* and *scxb* mRNA in *scxa* mutants shows reduced *scxa* signal in mutants compared with siblings, which is indicative of nonsense-mediated decay (Supplemental Fig. S2*D*). We observed no change in either the pattern or levels of expression of *scxb* in the *scxa^−/−^* mutants, which suggests that there is no compensatory up-regulation of *scxb* (Supplemental Fig. S2*E*). To investigate Scxa function, we imaged 7 days postfertilization (dpf) *scxa* mutants and siblings carrying a *col2a1a:mCherry* transgene by SHG microscopy, which reveals myosin heads in muscle and collagen (mainly collagen I) in tendons and ligaments (40), combined with confocal imaging of the transgene that labels cartilage. No obvious changes to cartilage were observed (**Fig. 2*A–C***). However, SHG revealed decreased signal in certain tendons and ligaments of *scxa* mutants, such as the sternohyoideus tendon, which is suggestive of poor collagen organization (Fig. 2*A, B*). To corroborate these results in *scxa* mutants, we performed *in situ* hybridization for *tnmd* at 3 dpf in an incross of *scxa^+/−^*. Genotyped *scxa* mutants (9/9) showed reduced *tnmd* expression levels in cranial tendons, whereas cleithrum expression was largely preserved (Fig. 2*D*). Using immunostaining for Tsp4b, a marker of tendons and ligaments (25), *scxa^−/−^* mutant embryos showed disorganization and changes to directionality and shape of cranial tendons and ligaments, which were variable between specific tendons and between individual mutants despite comparable levels of Tsp4b accumulation (8/8 of analyzed mutants, Fig. 2*E–G*). Muscle fibers visualized with MyHC in *scxa* mutants showed a range of abnormalities, such as fibers that extended beyond their normal boundaries, marked by Tsp4b, fibers that were misaligned or that crossed the midline, and disorganized junctions (Fig. 2*E–G* and Supplemental Fig. S3*A* and **Table 1**). Similar phenotypes were also seen in *scxa^kg141^* mutants (Supplemental Fig. S3*B*–*F* and Table 1) and occasionally in *scxa^+/−^* embryos, though at lower penetrance (Supplemental Fig. S3*A* and Table 1). We found no substantial *scxa* mutant phenotype in cartilage and bone by analysis of *Tg(col2a1:mCherry)* and alizarin red (AR)/AB staining at 6 and 13 dpf (Supplemental Fig. S4). Thus, lack of Scxa leads both to disruption of tendon and ligament morphology and to defects in the attachment and orientation of cranial muscle fibers.

**TABLE 1:** Analysis of head muscle phenotype in scleraxis mutants.

Phenotype*^a^*	+/+	*scxa^+/−^*	*scxa^−/−^*	*scxb^−/−^*	*scxa^−/−^ ;scxb^−/−^*
Intermandibularis anterior					
Overextension	0/10	0/14	1/8	0/5	1/4
Ectopic fibers	0/10	0/14	1/8	0/5	0/4
Muscle fibers misaligned	0/10	0/14	2/8	0/5	2/4
Intermandibularis posterior					
Overextension	2/10	5/14	8/8	2/5	4/4
Ectopic fibers	0/10	1/14	1/8	0/5	4/4
Muscle fibers crossing midline	0/10	1/14	6/8	1/5	4/4
Interhyoideus					
Overextension	1/10	5/14	5/8	0/5	4/4
Ectopic fibers	0/10	1/14	2/8	0/5	4/4
Muscle fibers crossing midline	0/10	2/14	5/8	0/5	3/4
Hyohyoideus					
Overextension	0/10	2/14	6/8	0/5	4/4
Ectopic fibers	0/10	2/14	2/8	0/5	4/4
Muscle fibers crossing midline	0/10	5/14	6/8	2/5	4/4
Sternohyoideus					
Overextension	0/10	0/14	0/8	0/5	0/4
Ectopic fibers	0/10	0/14	0/8	0/5	0/4
Muscle fibers crossing midline	0/10	1/14	0/8	0/5	1/4
Adductor mandibularis					
Overextension	0/10	1/14	4/8	0/5	0/4
Ectopic fibers	0/10	2/14	3/8	1/5	0/4

a Analyzed at 4 dpf in embryos from *scxa^+/kg170^*, *scxb^+/kg107^*, or *scxa^+/kg170^*;*scxb^+/kg107^* incrosses from immunohistochemistry of Tsp4b and MyHC (A4.1025).

### *Scxa* adult fish are viable but show reduced body size and muscle volume, as well as abnormal swim behavior

**Figure 3:**
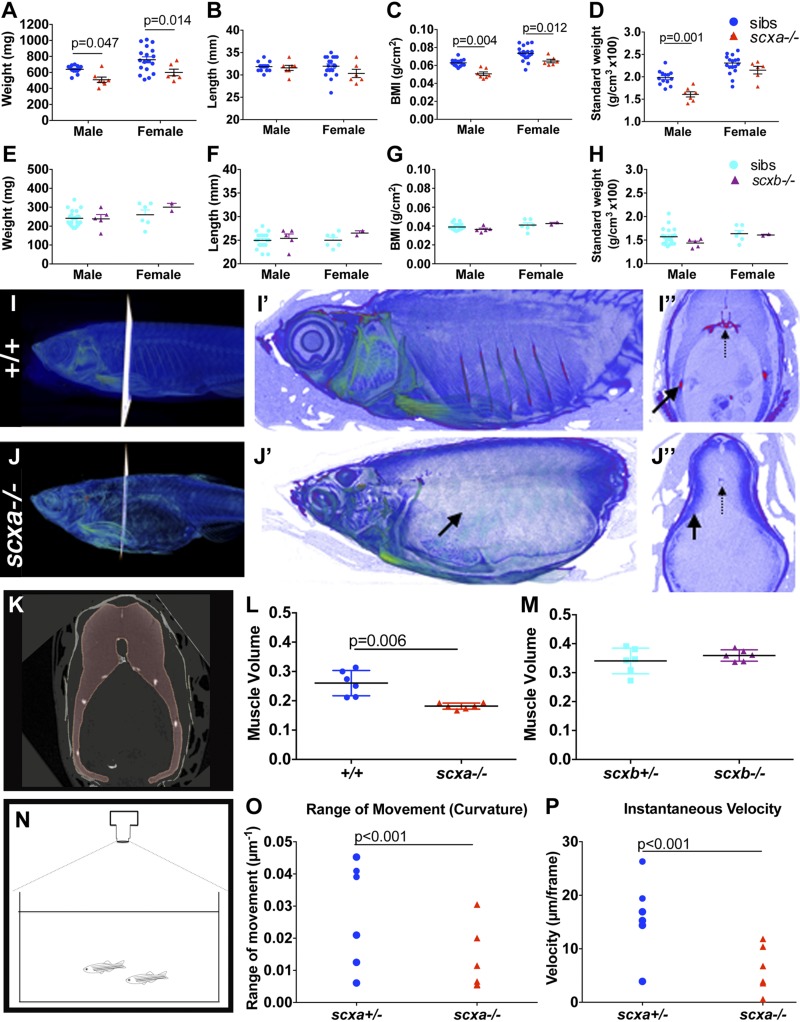
Lack of *Scxa* in adults affects body measurements and swim behavior. *A*–*H*) Weight, length, BMI, and standard weight comparison between mutants and sibling adult fish from a *scxa^+/−^* incross (13 siblings and 7 *scxa*^−/−^ males, 18 siblings and 6 *scxa*^−/−^ females, *A*–*D*) and a *scxb^+/−^* incross (21 siblings and 5 *scxb*^−/−^ males, 6 siblings and 2 *scxb*^−/−^ females, *E*–*H*). Males and females are presented separately as were found significantly different. *I*, *J*) Contrast-enhanced µCTs to show soft tissue of *scxa^−/−^* mutant (*J*–*J*″) and a ^+/+^ sibling (*I*–*I*″). Magnified scans show lack of ribs (black arrows) in the mutant. *I*″, *J*″) Transverse optical sections at indicated positions, where dashed arrows indicate the vertebrae. *K*) Myotome volume from co-reared similar-length fish are calculated from scans by creating a virtual steak between 2 ribs (red area, see Materials and Methods). *L*, *M*) Quantification of muscle volume in *scxa^−/−^* adult mutants (*L*) and *scxb^−/−^* (*M*) and their respective siblings. *N*–*P*) Swimming performance was calculated from videos taken from above the tank [schematic drawing in panel (*N*), see also Supplemental Video and Supplemental Fig. S7], and the range of movement (curvature, *M*) and instantaneous velocity (*N*) were calculated as detailed in the Materials and Methods. Two-way ANOVA statistics with Sidak’s *post hoc* tests were performed (*A*–*H*). Unpaired *t* test with Welch’s correction (*L*, *M*) and 2-way ANOVA (*O*, *P*). Significant *P*-values indicated on graphs.

Growth of zebrafish is dependent on feeding rate (52). To determine whether the cranial musculoskeletal defects in *scxa* mutants have consequences in later life, we examined growth of mutant fish and their siblings reared in the same tank. *scxa* homozygous mutants were viable and survived in relatively normal Mendelian ratios; 28 of 107 (26% mutants from the whole incross) adults from a heterozygote incross. Body measurements of 15-mo adult *scxa* mutant fish and their co-reared same-sex siblings showed that they weigh ∼15% less (**Fig. 3*A***). Overall body length was comparable between the different genotypes, suggesting that underfeeding was not the main cause for lower weight (Fig. 3*B*). Moreover, mutants also show a significantly reduced BMI and standard weight (*K*, Fulton’s body condition factor), measures that would compensate for any differences in overall growth rate (Fig. 3*C, D*). We analyzed contrast-enhanced µCT to allow visualization of soft tissue (Fig. 3*I*). Muscle volume is clearly smaller in mutants (compare Fig. 3*I, J*). Quantification of muscle volume from µCT scans (Fig. 3*K, L*) shows that adult *scxa* mutant fish have significantly lower (∼25% less) muscle volume than their cohabiting siblings (Fig. 3*L*). Nonmuscle volume did not differ between the groups (unpublished data). To test whether adult *scxa* mutant fish show altered swim behavior, we developed software to track videos of adult fish (Fig. 3*N*, see Materials and Methods). Both the range of movement (curvature) and instantaneous velocity of *scxa* mutants were significantly lower than those of co-reared heterozygotes (Fig. 3*O, P*). Thus, we conclude that fish lacking Scxa are thin and have reduced muscle and swimming ability compared with their siblings.

### *Scxa* adult fish lack rib mineralization and show bone growth and composition abnormalities

**Figure 4:**
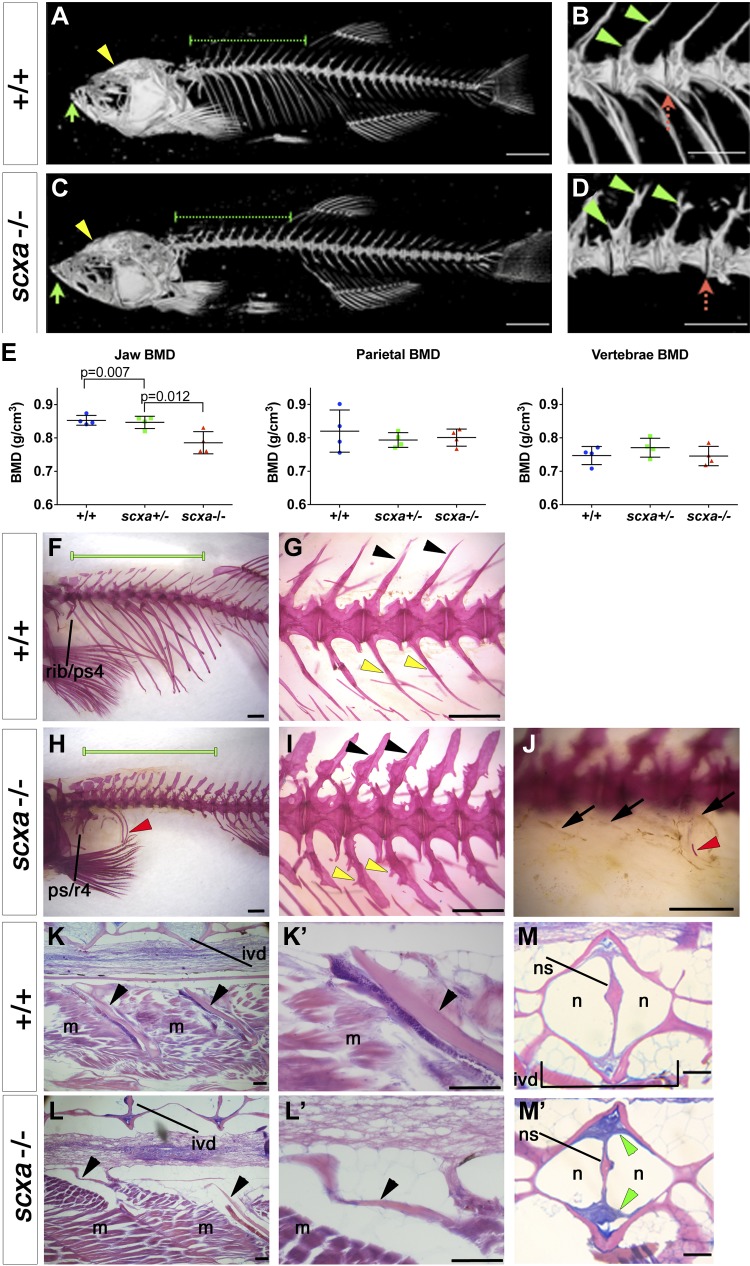
Adult *scxa* homozygous mutants have skeletal abnormalities. *A*–*E*) Three-dimensional volumetric isorenders from µCt data of *scxa^−/−^* and wild-type sibling showed absence of mineralized ribs (green dashed line above the rib region) (*C*, compared to *A*), and protruding jaws (green arrows) in mutants. Thoracic ribs region was magnified to show details of the vertebrae (*B*, *D*). Small bony structures were observed branching from the haemal arches (green arrowheads), and vertebrae misalignments were often present (red dashed arrow) in *scxa* mutants. *E*) BMD values were calculated from 3 distinctive bones: jaw (green arrows in *A*, *C*), vertebrae, and the parietal bone (yellow arrowhead in *A*, *C*). One-way ANOVA statistics with Tukey’s *post hoc* test performed, *P* values are as indicated. *F*–*J*) AR-stained adult *scxa^−/−^* mutants show no signal in ribs (*H*, under green line, compared with *F*), and neural (black arrowheads) and haemal arches (yellow arrowheads) have extensive bony growth (vertebrae 15–19 magnified in *G*, *I*). Fibrous, almost transparent ribs are seen in high magnification (black arrows, *J*), with the odd mineralized rib tissue (red arrowhead in *H*, *J*). The area rostrally to rib 5 is formed normally. Ps/r4, parapophysis and rib 4. *K*–*M*) Adult wild-type and *scxa* mutant zebrafish sagittal (*KK*′,*LL*′) and transversal (*MM*′) paraffin sections stained with H&E and AB. Existing rib structure in *scxa* mutant is short, thin, and wavy. The IVD is shown in *MM*′. The notochord string (ns) connecting the dorsal and ventral of the V shape is normal. However, the IVD is enriched with fibrous tissue (more purple, green arrowheads). M, muscle; n, notochordal cells. Scale bars, 1 mm (*A*–*D*, *F*–*J*), and 100 µm (*K*–*M*).

Because juvenile zebrafish express *scxa* mRNA near ribs and in other skeletal elements (Fig. 1), and our data from soft-tissue µCT showed missing ribs (Fig. 3*J*), we sought to investigate in detail the zebrafish *scxa* mutant skeleton. We performed µCT on 15-mo adult fixed fish (**Fig. 4*A–D***), which revealed a severe lack of rib growth and mineralization (see below) and multiple bony outgrowths from neural and haemal arches in the mutants (Fig. 4*B, D*). AR staining showed this phenotype in more detail (Fig. 4*F*–*I* and Supplemental Fig. S5*C, D*). We also used the µCT to test whether vertebral thickness differs between the groups; we extracted the centrum volume through image segmentation. However, no changes were observed, indicating that Scxa is not essential for vertebral bone thickness in zebrafish (Supplemental Fig. S5*A*, *C, D*). However, when the bony structure at the same anteroposterior level is calculated to include rib and arches, the mutants showed significant differences (Supplemental Fig. S5*A*, *C, D*). We calculated BMD in the lower jaw (dentary), the parietal skull, and vertebral centrae. Jaw BMD is significantly lower in *scxa* than in its siblings, whereas skull and vertebral BMD were unchanged (Fig. 4*E*). Thus, *scxa* mutants have a range of skeletal and cartilaginous abnormalities in both trunk and skull.

**Figure 5:**
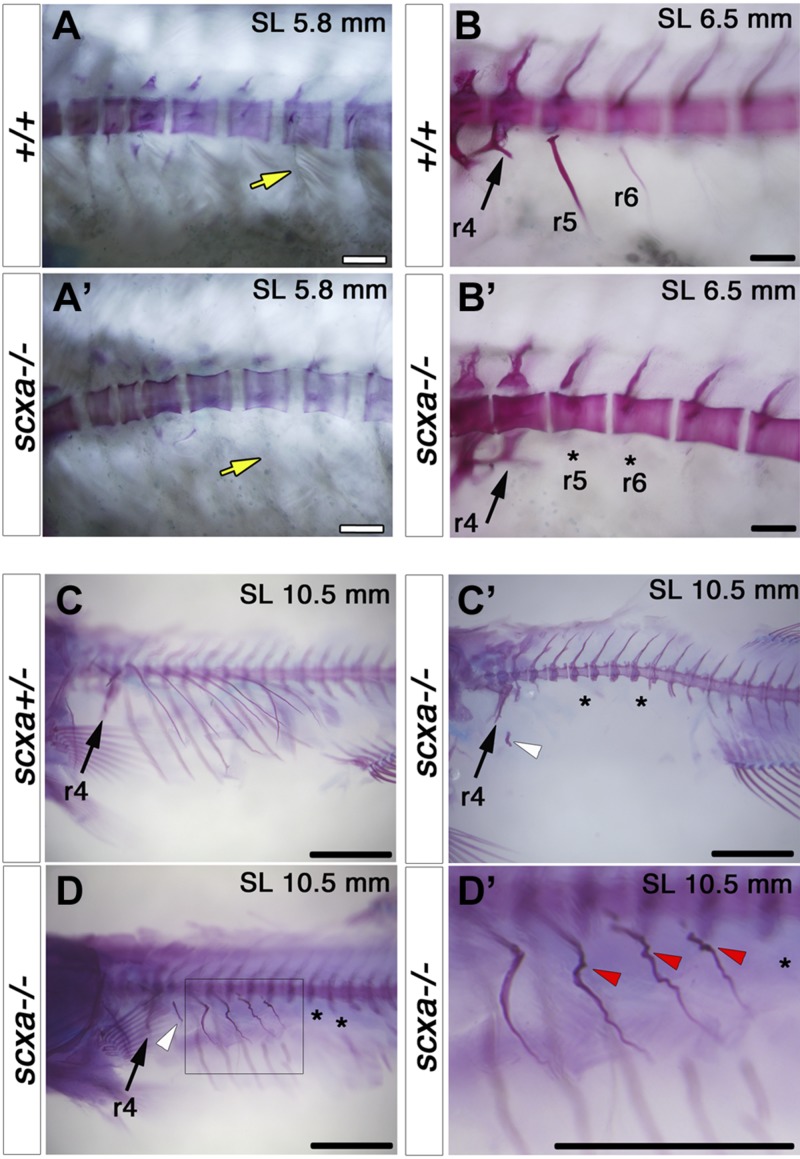
*Scxa* is is required for rib mineralization and its patterning. AR staining for fish from a *scxa^+/−^* incross at the indicated juvenile stages. *A*) At SL 5.8 mm, increased differential interference contrast (DIC) shows that the junction region between muscle and rib (yellow arrows) is already abnormal (*AA*′). *B*) At SL 6.5 mm, the first anterior ribs (r5 and r6) are lacking (asterisks in *B*′, compared with *B*). *C*) A severe lack of mineralized ribs (black asterisks) and mineralized rib fragments (white arrowhead) is seen at SL 10.5 mm (*C*′, compared with *C*). *D*) In another mutant (*D*), some ribs are missing (asterisks in *D*), whereas others are highly fractured and healed (r5–r9 magnified and marked by red arrowheads in *D*′). Note that r4 is formed normally (arrows in *B*–*D*). All scale bars, 100 µm.

The µCT also showed no mineralization in the thoracic ribs in 7/7 *scxa^−/−^* homozygous mutants, whereas 7/7 *scxa^+/+^* and 4/4 *scxa^+/−^* siblings had normal ribs (Fig. 4*A–D*). AR staining on similar age adults also revealed a lack of mineralization in ribs in 6/6 *scxa* mutants, whereas 7/7 *scxa^+/+^* showed normal ribs (Fig. 4*F–I*). Most juvenile mutants stained with AR also lacked mineralization (7 of 9 analyzed mutants), whereas the remaining 2 showed a milder phenotype with uneven mineralization in which some ribs were missing and others displayed residual mineralization (see **Fig. 5**). No siblings lacked mineralization (0 of 22 analyzed siblings). However, close examination of mutants that lacked mineralization revealed that some unstained, glossy, rib tissue was present (Fig. 4*J*), suggesting that Scxa is required for normal mineralization or growth, rather than formation of ribs *per se*. Indeed, H&E combined with AB staining on adult sections unveiled rib-like structures between the myotomes (Fig. 4*K, L*). This histologic analysis also revealed an accumulation of fibrous tissue in the intervertebral disk (IVD), suggesting abnormality with the notochord sheath cell layer (Fig. 4*M*).

### *Scxa* mutants have abnormal rib mineralization from patterning stage

To better understand the nature of the rib growth and mineralization defect, we stained juveniles with AR and AB at various stages around the time when ribs are formed. Ribs develop anteriorly to posteriorly beginning at 5.8 mm SL, as seen by AR or Calcein staining (53, 54). At juveniles of SL 5.8 mm, although no mineralized ribs were detected in any genotype, the arrangement of the vertical myoseptum and the rib region appeared abnormal in the mutants (Fig. 5*A*; 2 of 2 *scxa* mutants analyzed). Juveniles reaching SL 6.5 mm had initiated mineralization in the anterior ribs (ribs 5 and 6), but *scxa* mutants at that stage lack mineralized ribs. However, the Weberian ossicles located anterior to the ribs developed normally (Fig. 5*B*; 4 of 4 larvae examined). In SL 10.5-mm juveniles, ribs had formed in siblings (Fig. 5*C*), but *scxa* mutants either lacked mineralized ribs (Fig. 5*C*; 3 of 5 mutants analyzed) or showed a milder phenotype with some ribs missing and others small, bent, and twisted (Fig. 5*D*; 2 of 5 mutants analyzed) as occasionally found in adults (Supplemental Fig. S5*E*). These defective ribs are reminiscent of the ribs in mutants in which altered collagen composition causes weak and bent ribs that may reflect a history of repeated fracture repair (55). These observations show that *scxa* mutants have defects in the tendon-like regions between the myotomes where ribs form already at the patterning stage rather than it being a remodeling in response to later events.

### *Scxa* mutants maintain normal MTJs in the somites

**Figure 6:**
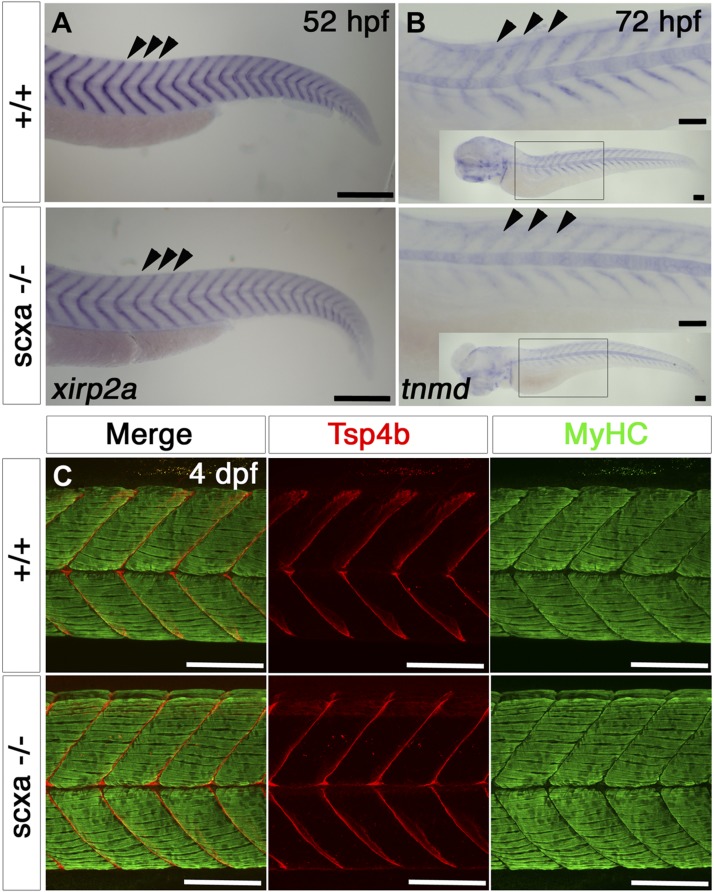
*Scxa* mutants have reduced levels of tendon markers, but somitic MTJs appear normal. *A*, *B*) *In situ* hybridization for *xirp2a* at 52 hpf (*A*) and *tnmd* at 3 dpf (*B*) for *scxa^−/−^* and their siblings (+/+), lateral view, anterior to left. *B*) The main image shows the anterior somites, inset-whole embryo. Both *xirp2a* and *tnmd* mRNA levels are reduced at the MTJs at the somitic borders (arrowheads). *C*) Confocal stacks of immunodetection of MyHC (A4.1025) and Tsp4b in somites 11–14 of 4 dpf embryos of *scxa^−/−^* and siblings showing normal distribution of Tsp4b and normal muscle structure. All scale bars, 100 µm.

During development, muscle fibers from each side of the vertical myoseptal somite border align, connect, and secrete ECM proteins that create a distinctive extracellular MTJ structure. Anchorage to this MTJ allows fibers to transmit and withstand contraction forces (reviewed in ref. 56). Many tendon markers are expressed at the MTJ either by the neighboring muscle cells or by fibroblast-like cells proposed to be tenocytes (22). Indeed, by 4 dpf we observed fibroblast-like cells with the matrix protein Tsp4b surrounding their nuclei and with long processes extended into the myosepta (Supplemental Fig. S6*A*). Given that *scxa* is expressed early in the MTJ (Fig. 1*A* and ref. 23), we tested whether defective early MTJ patterning might underlie the rib and muscle defects. *In situ* hybridization for *xirp2a* mRNA, a marker for somitic MTJ, for embryos from a *scxa^+/−^* incross at 52 hpf showed reduced expression in 9 of 9 genotyped scxa mutants (**Fig. 6*A***) but not in siblings (17/17). At 72 hpf, *tnmd* mRNA, a marker for maturing tenocytes, was also decreased in *scxa* mutant larvae (9 of 9 genotyped mutants) but not in siblings from a similar cross (Fig. 6*B*). In contrast, immunohistochemistry for the Tsp4b protein, which controls matrix assembly in the MTJ (25), yielded no detectable change in level or distribution in *scxa* mutants (Fig. 6*C* and Supplemental Fig. S6*B*; 5 of 5 analyzed mutants). We conclude that some but not all MTJ markers are reduced in *scxa* mutants.

To determine whether MTJ defects were secondary to muscle defects at these early stages, *scxa* mutants were bred into the transgenic *Tg(actc1b:egfp)^zf13^*. Somite muscle architecture appeared normal as seen by MyHC, α-actinin, and green fluorescent protein distribution (Fig. 6*C* and Supplemental Fig. S6*D*). The ECM protein laminin and the fiber-end–associated cytoskeletal link protein dystrophin also did not distinguish mutants from their siblings (Supplemental Fig. S6*D* and unpublished data). Thus, early myogenesis appears normal in *scxa* mutants.

### *Scxb* mutants are viable and show no obvious defects

We also examined *scxb* homozygous mutants at embryonic, juvenile, and adult stages. During embryonic stages, *scxb* mutants show neither tendon or muscle craniofacial abnormalities (Fig. 2*C* and Supplemental Fig. S3*G* and Table 1) nor difference in AR/AB staining at 6 dpf (unpublished results). Somitic MTJ of *scxb* mutant embryos also developed normally as seen by normal Tsp4b distribution in the myosepta (5 of 5 mutants and 13 of 13 genotyped sibling larvae), and somitic muscle volume and structure was unchanged (MyHC; Fig. 3*M* and Supplemental Fig. S6*C*). Adult *scxb* mutants are viable and survive in normal numbers (9 of 39; 23% mutants from a heterozygote incross). Measurement of 4- and 12-mo-old *scxb* mutants showed no significant differences between *scxb* mutants and siblings in weight, length, BMI, and standard weight (Fig. 4*D*–*F* and unpublished results). The skeleton was evaluated by µCT (3 of 3) and AR (4 of 4, Supplemental Fig. S5*F*), and the mutants were shown to be normal and indistinguishable from siblings (Supplemental Fig. S5*F*, *G* and unpublished results). Thus, the lack of Scxb has no detectable effect on the development of the zebrafish musculoskeletal system.

### Lack of Scxa and Scxb leads to lethal jaw defects

**Figure 7:**
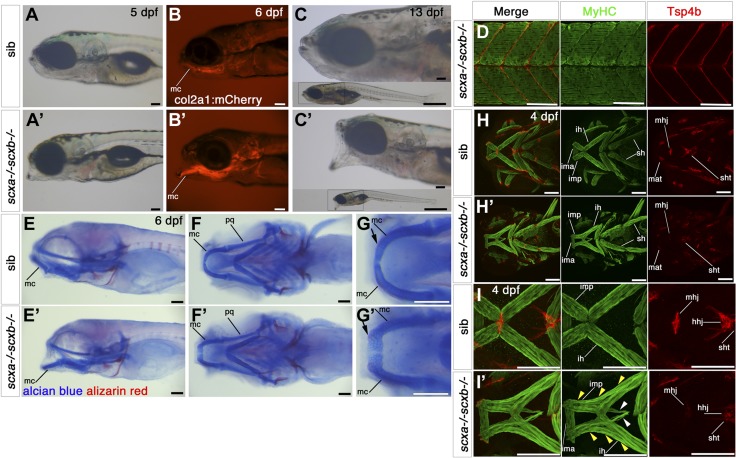
*Scxa;scxb* double mutants have a jaw phenotype and severe musculoskeletal defects. *A*–*C*′) Live transmitted light and red fluorescent images in lateral view, anterior to left of genotyped *scxa^−/−^;scxb^−/−^* double mutants (*A*′–*C*′) compared with siblings (*A*–*C*, shown is *scxa^+/−^scxb^+/−^)*. Mutants show a hanging open jaw. The *col2a1:mCherry* transgene (*B*) shows the dropping Meckel’s cartilage (mc). At 13 dpf, fish are much smaller than siblings (whole-fish insets in *C*). *D*) Confocal stacks of immunodetection of MyHC (A4.1025) and Tsp4b in 4 dpf embryos of *scxa^−/−^ scxb^−/−^* showing normal distribution of Tsp4b and normal muscle structure (compare with Fig. 6*C*). *E*–*G*′) AB/AR staining for cartilage and bone for *scxa^−/−^scxb^−/−^* (*E*′–*G*′) and sibling from the same cross (*E*–*G*) shown in lateral (*E*) and ventral (*F*, *G*) views that show the hanging jaw phenotype. The magnified area from mc shows many less differentiated rounded cells at its most anterior tip, near the joint (arrow, *G*′) compared with elongated mature cells in sibling (arrow, *G*). *H*–*I*′) Confocal stacks showing immunodetection of cranial muscles (MyHC, A4.1025) and tendons (Tsp4b) of 4 dpf embryos of *scxa^+/−^*; *scxb^+/−^* incross in ventral view. Tsp4b is highly reduced in tendons and ligaments of double mutants as shown for mandibulohyoid junction (mhj) (magnified area, *II*′). Many muscle fibers in *scxa*^−/−^; *scxb^−/−^* extend the length of the intermandibularis posterior (imp) and the interhyoideus (ih) (yellow arrowheads, *I*′), whereas others extend far beyond their normal end at the mhj until their meeting point (white arrowheads, *I*′). All scale bars, 100 µm. Hhj, hyohyoideus junction; ih, interhyoides; ima, intermandibularis anterior; imt, intermandibularis tendon; mat, Meckel’s adductor tendon; mc, Meckel’s cartilage; pq, palatoquadrate cartilage; sh, sternohyoideus; sht, sternohyoideus tendon.

We generated double mutants from incrosses of *scxa^+/kg170^;scxb^+/−^*. Double mutants were indistinguishable from their siblings before 4 dpf and were viable in normal Mendelian numbers up to early juvenile stages (7 of 138 from total number of genotyped embryos at 6–13 dpf, χ^2^ test *P* = 0.567). Immunostaining for Tsp4b and MyHC showed no phenotypic abnormality in the MTJ (**Fig. 7*D*** compared with Fig. 6*C*), showing that *scxa* and *scxb* combined function is not essential for early somite MTJ development.

In embryos from incrosses of *scxa^+/kg170^;scxb^+/−^*, a subset had abnormal lower jaw, which hung open from 4 dpf, (Fig. 7*A, B*). This phenotype showed in 22 of 533 embryos (4.1% of all embryos), of which there were *scxa^−/−^;scxb^−/−^* (16 of 22) and *scxa^−/−^;scxb^+/−^* (6 of 22, ∼10% penetrance). No normal embryos (25 genotyped) were double mutant, but 1 embryo was *scxa^−/−^;scxb^+/−^*. The jaw morphology defect caused reduced jaw movement, although they were motile and able to swim (unpublished results). At 13 dpf, *scxa^−/−^;scxb^−/−^* larvae had a similar jaw phenotype and severe growth retardation (3 of 45, 6.6% from all embryos; Fig. 7*C*), and by 34 dpf, no surviving *scxa^−/−^;scxb^−/−^* fish were obtained (0 of 41, 0% from all embryos). This was confirmed by an incross of *scxa^+/kg141^;scxb^+/−^* that yielded no double mutants (0 of 63, 0% from whole surviving incross, tested at 12 mo of age). Overall, lack of *scxa^−/−^;scxb*^−/−^ fish is significant (χ^2^ = 0.008), indicating that they die in the period between 2 and 5 wk of age. *scxa^−/−^;scxb^+/−^* fish were found in expected Mendelian numbers for both incrosses.

To further investigate the hanging jaw phenotype, we stained 6 dpf larvae from an incross of *scxa^+/kg170^;scxb^+/−^* for AR/AB (Fig. 7*E–G*). *scxa^−/−^;scxb^−/−^* fish show a clear change of position of the Meckel’s cartilage (Fig. 7*B, E*). The joint at the anterior tip of the Meckel’s cartilage contains many rounded cells that are undifferentiated as opposed to elongated mature cartilage in siblings (compare Fig. 7*F,G*). All double mutants (6 of 6) showed a severe reduction in Tsp4b and disorganization in cranial tendons and ligaments, most severely in the mandibulohyoid junction between 4 and 13 dpf (Fig. 7*I*). Functional muscle has been shown to be required for normal jaw morphology (57, 58). We found that *scxa^−/−^;scxb^−/−^* larvae had more severe defects in jaw muscles, particularly in the intermandibularis posterior and interhyoideus muscles. A large proportion of the intermandibularis posterior muscle fibers from either side of the midline extended beyond the mandibulohyoid junction, where they normally end, until their displaced meeting, whereas others appeared to change angle and also be part of the interhyoideus muscle (compare Fig. 7*H, I* and Table 1). Thus, lack of both Scx genes in zebrafish cause substantial tendon, muscle, and cartilage defects that result in paralysis of the jaw with lethal consequences.

## DISCUSSION

The findings described here provide genetic demonstration of 3 major points. Firstly, that *scxa* is required for correct skeletal development, including rib growth and mineralization, morphology of vertebral arches, normal swimming behavior, and trunk muscle composition. Secondly, *scxa* mutation leads to embryonic defects in cranial tendon formation and muscle misalignment. Thirdly, whereas loss of *scxb* alone does not lead to severe phentoypes, *scxb* is required in the absence of *scxa* because loss of both leads to lethal jaw paralysis. From this we conclude that *scxa* and *scxb* have overlapping functions in tendon formation.

### *scxa* and *scxb* expression and function during embryonic stages

In addition to confirming the expression of *scxa* in the craniofacial tendon precursors and the MTJ (23, 24), we demonstrate that *scxa* mRNA is expressed in the full extent of the vertical myosepta at juvenile stages. Loss of Scxa affects the structure and shape of various tendons, most notably ones connecting the jaw muscles to the jaw. This leads to abnormal connections, ectopic growth, and misalignment of fibers of the craniofacial musculature. *scxa* mutants are viable, however, surviving to adulthood despite a range of phenotypes. Although craniofacial cartilage and bone appear normal in embryos, our µCT data show significantly lower BMD in jaws of adult *scxa* mutants. The jaw bones mineralize much later than in our larval analyses. This may reflect changes to muscle activity that is due to the above tendon and muscle phenotypes leading to changes in the mechanical load of certain areas. Mechanical loading has been previously shown in humans and fish to affect bone properties, specifically BMD (59, 60). We show that *scxb* is expressed in a subset of cranial tendons, and weakly in the intersomite MTJs. Loss of Scxb alone led to no obvious phenotype in the presence of wild-type Scxa. However, mutation of *scxb* in *scxa* mutants exacerbates the phenotype, such that muscles are misaligned, extend into different muscles, and lack attachment to the skeleton. This leads to jaw paralysis, resembling the flaccid paralysis observed upon treatment with tricaine mesylate (58). We observe growth retardation and death in the early juvenile stages, likely from starvation. We, and others, have previously shown that periods of larval immobility affect the formation of jaw skeletal elements and joints (57, 58, 61, 62). Similarly, we show that tendon malformation can affect the muscle and the morphogenesis of the jaw, indicating synergy in development of the cranial musculoskeletal system. Some of the defects are reminiscent of the zebrafish *cyp26b1* mutant tendon phenotypes (24). In *cyp26b1* mutants, tenoblasts fail to condense into nascent *scxa*-expressing tendons, affecting muscle projection and misdirecting it. Our data show that scx function in tenoblasts contributes to the maturation of cranial tendons. None of the described mouse loss-of-function models reported head phenotypes (11, 14). However, *Scx* is expressed in pharyngeal arches and facial tendons of mouse embryos (8–10), and other mutations affecting mouse tongue muscle tendons resulted in tongue muscle abnormalities and dysmorphogenesis (63, 64). Facial tendons in mice, chicks, and zebrafish have a common origin from cranial neural crest cells (8, 23, 27). They are also similar in function, although different fish, birds, and mammals have varied feeding strategies and mandibular morphology (65, 66) such that cranial phenotypes stemming from tendon defects may differ between species.

The role that Scxa and Scxb has in the somitic MTJs seems to be limited at embryonic stages. We detected mild down-regulation of tendon markers and downstream targets, such as *tnmd* and collagens, which is comparable with mammals and birds (11–14). However, we found no evidence for down-regulation of key components of the ECM, such as thbs4b and laminin and functional embryonic phenotype, neither did we detect damaged muscle, loss of sarcomeric structure, or somitic boundary compromise in either the single or double mutants at larval stages. This contrasts with the phenotype observed upon loss of the dystrophin-associated glycoprotein complex components (reviewed in refs. 56, 67). This may reflect low levels of expression of *scxa* and *scxb* at embryonic stages, but other genes, likely in the fibers themselves, could control these ECM components at the somitic MTJs. It suggests that the muscle-dependent dystrophin-associated glycoprotein complex and integrin complexes are independent from tendon development and are sufficient to connect the somitic muscle blocks even when the tendons are impaired, thus preventing damage at least during embryonic stages.

### Scleraxis function in rib mineralization

Our data show that Scxa is strongly expressed in the intramuscular tendons adjacent to the developing ribs and is essential for rib growth and mineralization. In *scxa* mutants, we see severe defects in rib structure, such that mutants lack mineralized ribs. The tissue appears to become fibrous rather than bony. *scxa* mutants display changes to the structure of the vertebral arches, which are wide and irregular despite the normal myotome patterning seen at larval stages.

Many studies have indicated that all parts of the ribs are derived from the sclerotome compartment of the somites (68–71), whereas other studies have suggested that the ribs can be divided into 3 regions and that rib development also depends on the dermomyotome (72, 73). In addition, manipulations in chicks, which led to loss of Scx expression, such as separating the ectoderm physically from somites or changes to MKP3 levels affecting ERK signaling strength, resulted in defective distal rib development (74, 75). Although in mice *Scx* is expressed in rib primordia (8, 11, 13), the ribs and the tendons that connect them to the intercostal muscles were unaffected in 1 *Scx*^−/−^ allele (11), and the rib cage was decreased in size for the *Scx^cre/cre^* allele (14). Conditional inactivation of *Sox9* in *Scx^+^Sox9^+^* cells in *ScxCre;Sox9^flox/flox^* mice, caused a loss of all but the proximal rib cage (76). Mammalian tendon-bone attachments, including the patella, deltoid tuberosity, olecranon, and other eminences, express both *Sox9* and *Scx* (16, 76, 77). It is unclear if *scxa* is expressed in rib precursors in fish and whether it has a similar role in mineralization of the ribs as in mineralization of these mammalian eminences (11, 14, 16, 17, 76, 77). Function may also be maintained in the distal parts of the ribs in some amniotes. Ribs protect internal organs in fish and amniotes. The development of lungs and the requirement to protect the respiratory system with a strong bony rib cage may have shifted rib development to be more dependent on *Sox9* in land-living animals, and so affecting rib composition and strength.

Interestingly, we found that zebrafish lacking rib bone are viable, but their swimming performance is altered. This may indicate that the ribs play a mechanical role in swimming. Indeed, some studies have highlighted correlations in intramuscular ossification in fish that differ in their swimming modes (78, 79). The reduced volume of trunk musculature and body weight in *scxa* adult mutants could be linked to the altered swim behavior seen in the mutants. This, in turn, could be due to musculoskeletal attachments to the ribs and other intramuscular bones between the myomeres that are not capable of transmitting the full force from muscle contractions. Alternatively, altered intramuscular attachments in the trunk or abnormal fin attachments could alter both swim performance and preclude rib development. Although *scxa;scxb* double mutants are smaller in length and have paralyzed jaw, both linked to reduced feeding, *scxa* mutants have normal standard length, and their jaw movement seemed normal. However, we cannot rule out the possibility that reduced feeding affects the lower weight of *scxa* mutants, which in turn may be due to the mutants being outcompeted by their siblings.

Both our expression and functional data points to a greater role for *scxa* than for *scxb* in both head and trunk development, especially at juvenile stages. This likely reflects the high synteny of *scxa*, but not *scxb*, to the mammalian orthologs, likely keeping ancestral regulatory elements intact. However, Scxb protein is very similar to Scxa protein: a 70.8% identity between the 2 proteins, almost identical bHLH domain (Supplemental Fig. S2*A*, *B*), overlapping expression, and the ability to replace the function of the other to some extent.

In summary, we have shown that Scx has an essential role in the normal development of the musculoskeletal system in fish. Its essential function in differentiation and maturation of tendons, and in ossification of skeletal elements that express Sox9 and Scx, are conserved with other vertebrates. Zebrafish are thus a useful model to study the close relationship of muscle, tendon, and bone in development and disease of joints.

## Supplementary Material

This article includes supplemental data. Please visit *http://www.fasebj.org* to obtain this information.

## Abbreviations

µCT: microCT
AB: alcian blue
aa: amino acid
AR: alizarin red
bHLH: basic helix-loop-helix
BMD: bone mineral density
BMI: body mass index
bp: base pair
CRISPR: clustered regularly interspaced short palindromic repeats
Cas9: CRISPR-associated protein 9
CT: computed tomography
dpf: days postfertilization
ECM: extracellular matrix
H&E: haematoxylin and eosin
hpf: hours postfertilization
IVD: intervertebral disk
MTJ: myotendinous junctions
MyHC: myosin heavy chain
scx: scleraxis
SHG: second harmonic generation
SL: standard length
tnmd: tenomodulin
