# Supplementary material for: Scleraxis genes are required for normal musculoskeletal development and for rib growth and mineralization in zebrafish
Source: FASEB J. 2019 May 17;33(8):9116–30. doi: 10.1096/fj.201802654RR (PMC6662971; doi:10.1096/fj.201802654RR)
Supplement: Supplementary file 1 [file fj.201802654RR.sf1.pdf]

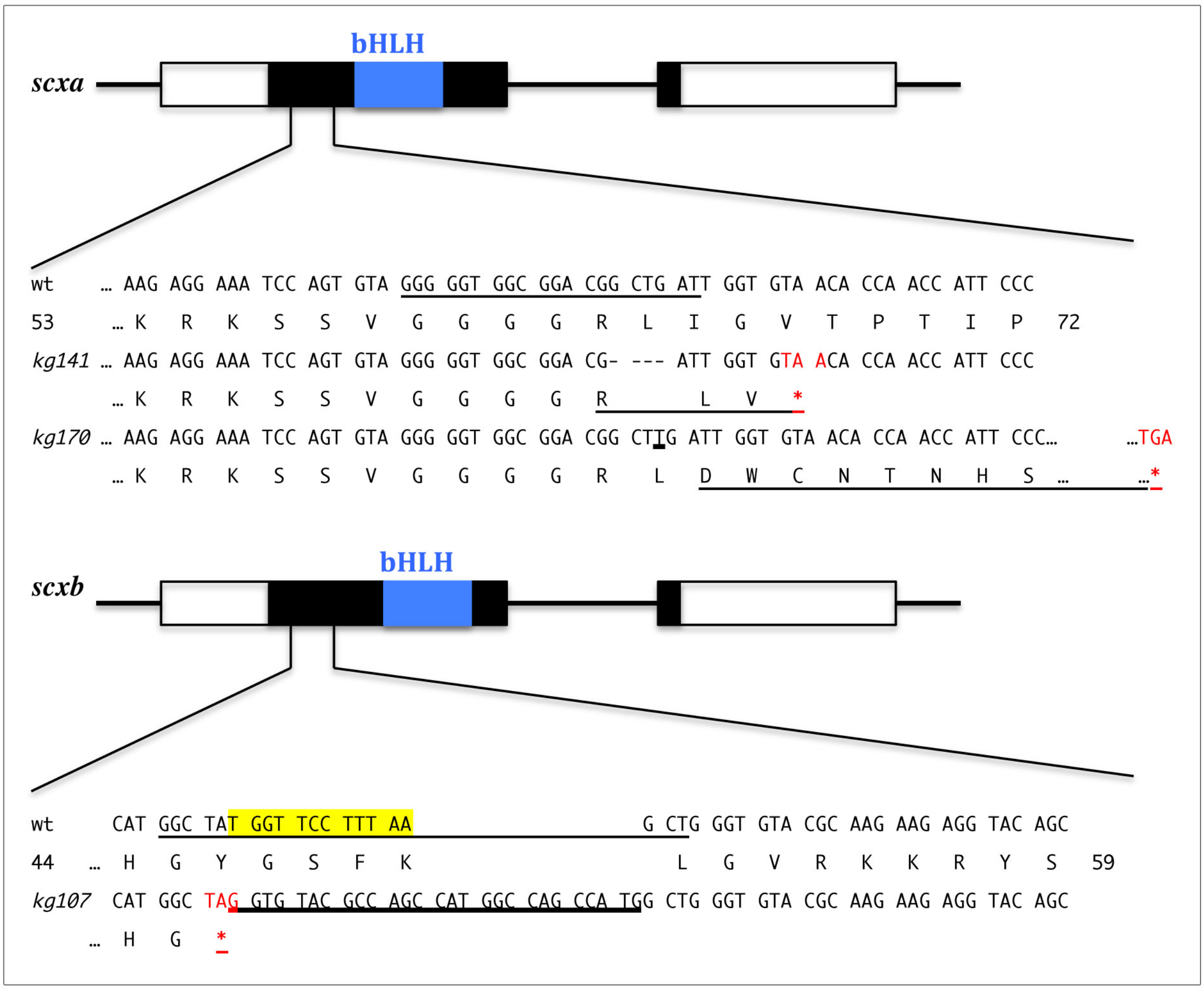

Figure S1. Generating mutant alleles for *scxa* and *scxb*. A. Schematic representation of *scxa* and *scxb* gene and protein and the new mutant alleles for *scxa (kg141* and *kg170)*, and *scxb* (*kg107)*. Each gene has two exons (Coding sequence shown by filled boxes, UTRs-white boxes, introns are marked with a black line). All mutations produce truncated proteins devoid of the basic and helix–loop–helix domains (bHLH, light blue). Beneath, DNA and protein sequence of wild type (wt) and mutant alleles are shown. CRISPR target sequence is underlined in the wt DNA sequence. Deleted nucleotides are shown by (-) in mutants or highlighted in the wt sequence and inserted nucleotides are shown with thick underscore. Presumed stop codons are shown in red text. Amino acid (aa) tails after frameshifts are underlined (only shown partially for *kg170*).

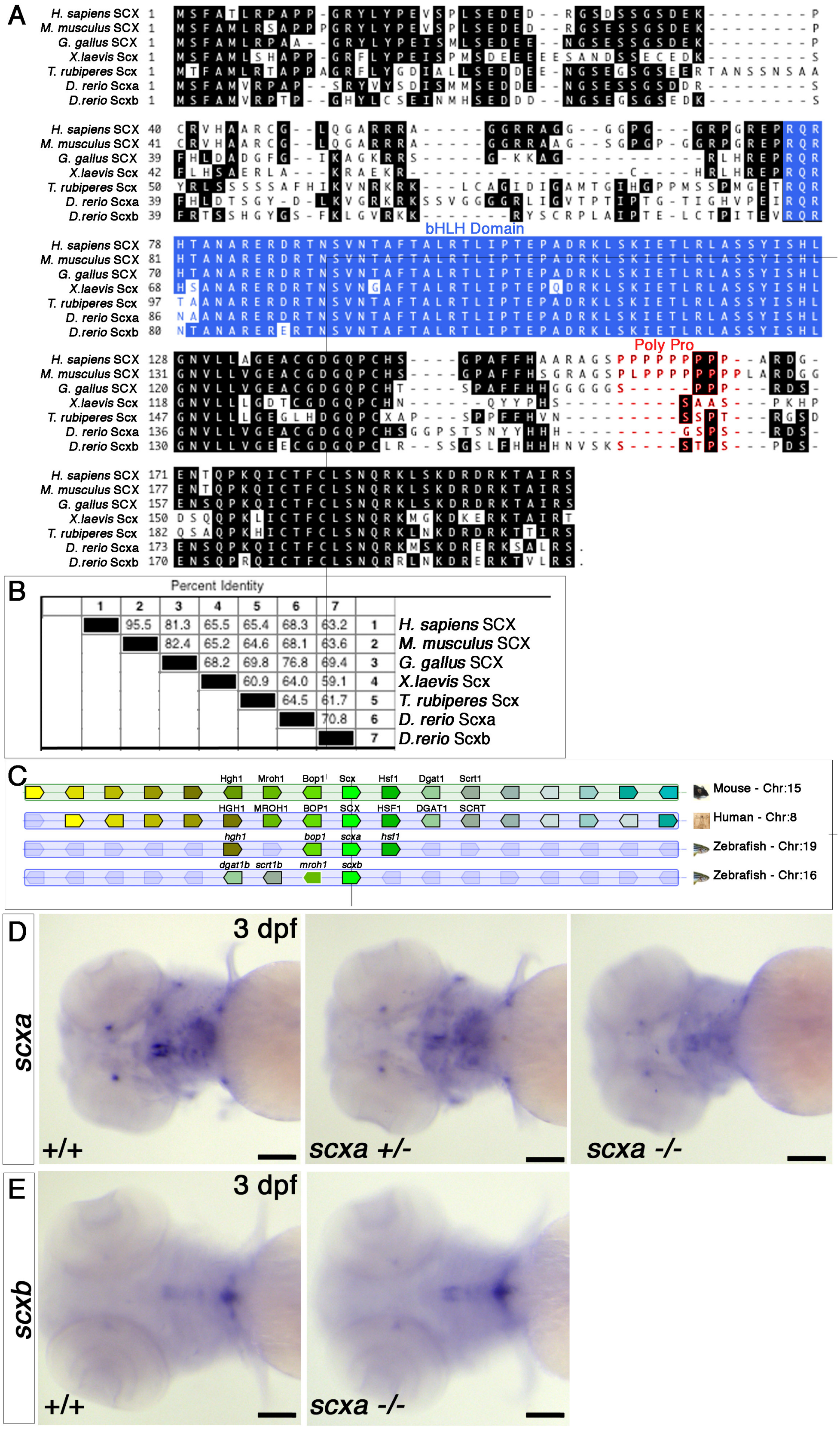

Figure S2. Scxa is the more conserved homologue of the mammalian Scx.
A. Clustal alignment of the translation product of the zebrafish *scxa* and *scxb* genes with representatives of other major vertebrate groups (human, mouse, chicken, *Xenopus laevis* and *Fugu rubripes*). **B.** Sequence pair distances of representative the above proteins using the Clustal method with PAM 250 residue weight table. **C.** Synteny diagram based on Genomicus software and Ensembl GRCz11 showing similar position of *scxa* gene inside intron 3 of the Bop1 gene on the other strand, as described for mouse *Scx* (11), whereas *scxb* locus show changes and rearrangements compared with mouse and human genomes. **D-E.** In situ hybridisation for *scxa (D)* and *scxb (E)* for 3 dpf embryos from a *scxa*$^{+/-}$ incross. Cranial expression is shown in ventral view. *scxa* mRNA levels in *scxa* mutant (11/50 embryos from the incross) are reduced compared with heterozygoute (29/50) and +/+ embryos (10/50), genotypes confirmed (D). *scxb* mRNA levels and spatial pattern do not differ between genotypes, genotypes confirmed (E). scale bars-100µM.

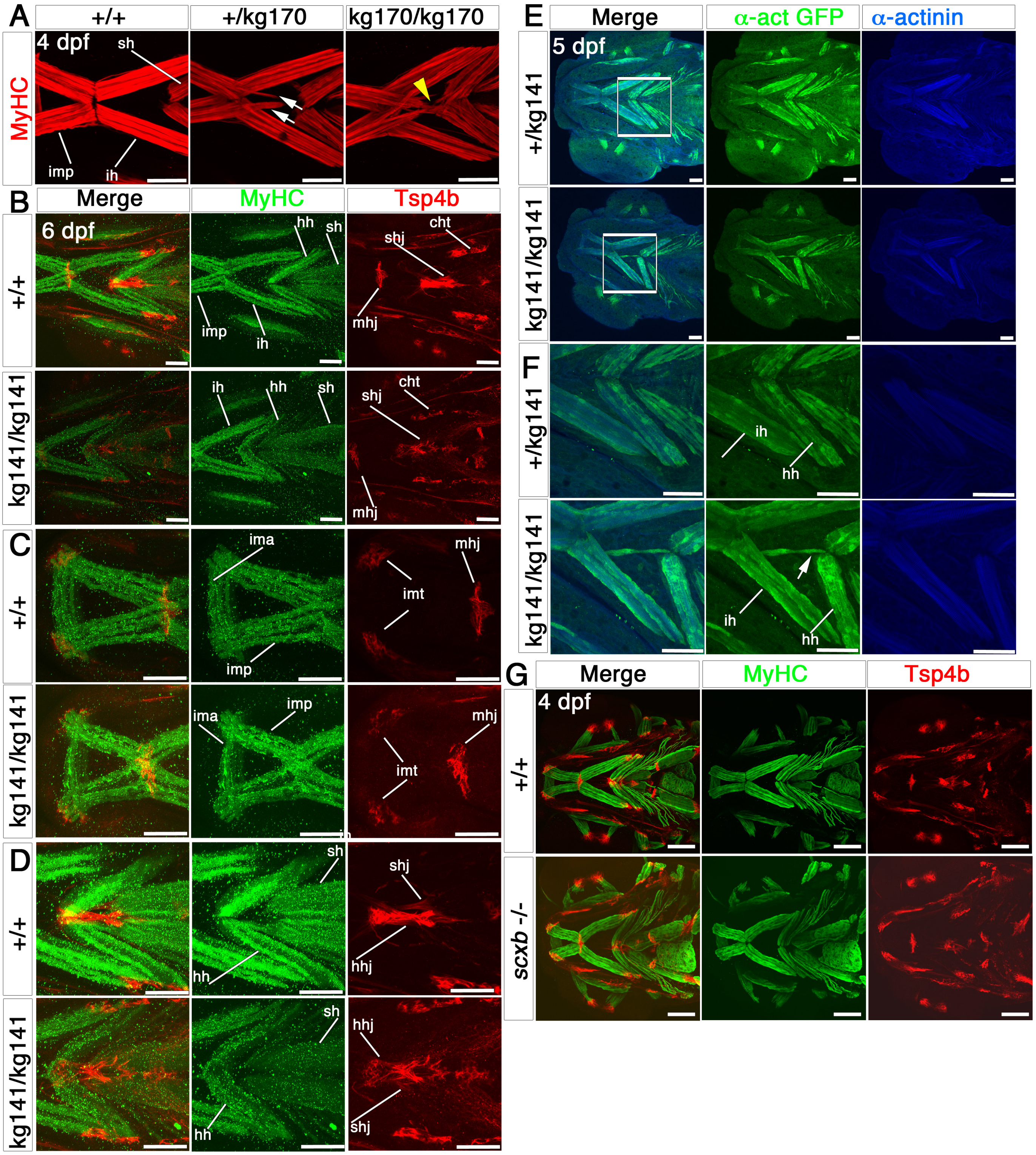

Figure S3. Cranial tendons, ligaments and muscles of *scxa*^{kg141} mutants are abnormal and disorganized. Confocal stacks of cranial muscles using immunofluorescence for MyHC (A4.1025, red in A), Tsp4b (red) and MyHC (MF20, green) in B-D, GFP (green) and alpha-actinin (blue) in E,F and Tsp4b (red) and MyHC (A4.1025, green) in G. All in ventral view, anterior to left. **A.** Embryos from a *scxa*$^{kg170/+}$ incross at 4 dpf; *scxa*$^{-/-}$ mutants had misaligned fibres and tri- and four-way abnormal junctions (yellow arrowhead). Some *scxa*$^{+/-}$ embryos had some milder defects (white arrows). **B-D.** 6 dpf embryos from a *scxa*$^{kg141/+}$ incross*;* the matrix protein, Tsp4b was downregulated in mutants, and some tendons such as the mandibulohyoid junction and intermandibular tendon (C), the sternohyideus and hyohyoideus tendons (D) were misshapen and showed decreased matrix condensation. Muscle fibre defects contained fibres connecting to wrong muscles and disorganized junctions. **E,F*.*** 5 dpf embryos from a *scxa*$^{kg141/+}$*;Tg(actc1b:egfp)*^{zf13} incross. F shows magnified boxed region in E. Mutant embryos had fibres from the interhyoideus muscle growing in the wrong direction towards the hyohyoideus junction (white arrow). **G.** Both tendons and muscles of 4 dpf embryos from a *scxb*$^{+/-}$ incross looked normal. mhj, mandibulohyoid junction, ima, intermandibularis anterior, imp, intermandibularis posterior, imt, intermandibular tendon, sht, sternohyoides tendon, hhj, hyohyoideus junction, ih, interhyoideus, hh, hyohyal, sh, sternohyoides. All scales 100µm except A, 50µm.

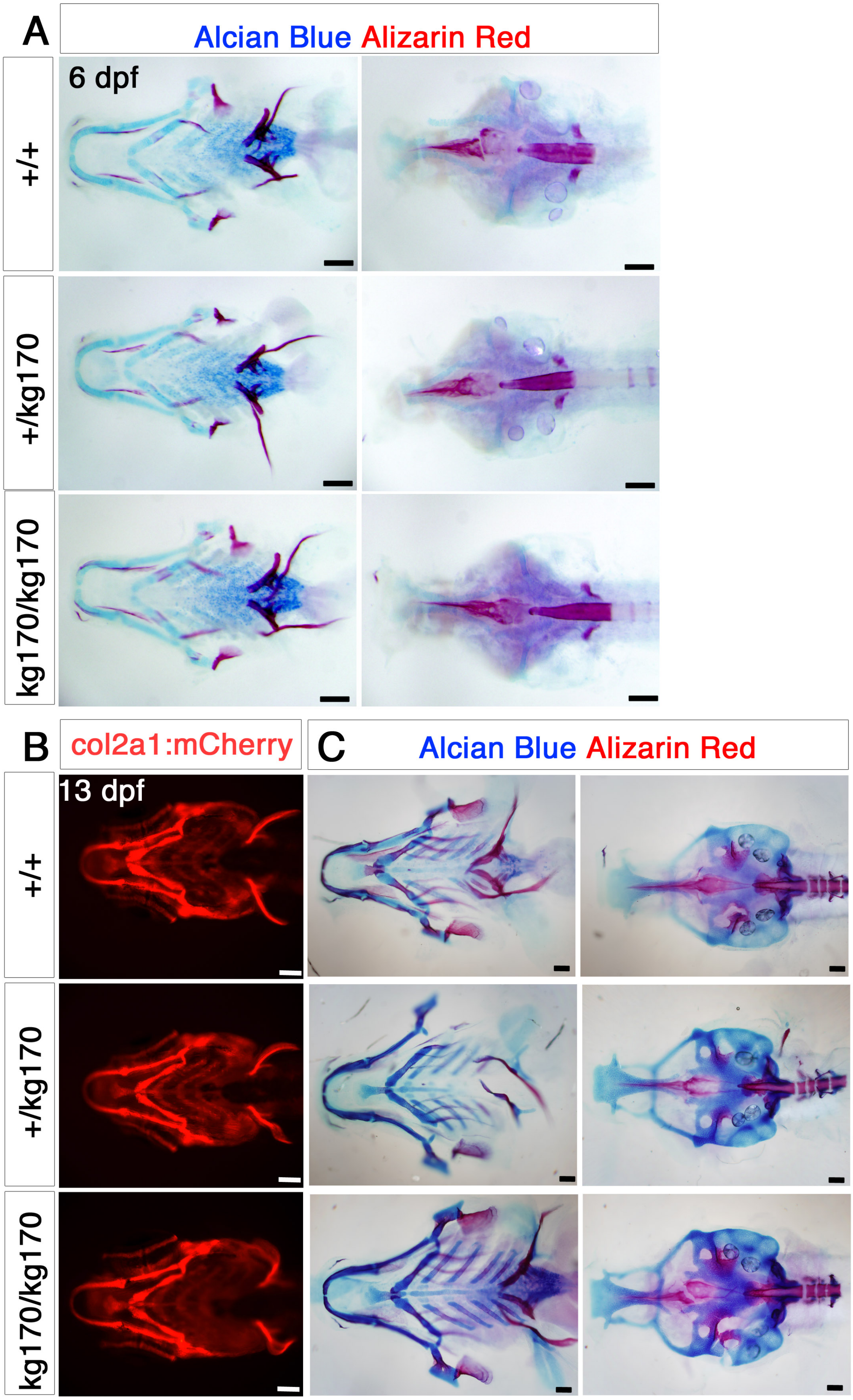

Figure S4. Cranial bone and cartilage show no obvious defects at embryonic and juvenile stages. A. Alcian blue and Alizarin red double staining for cartilage and bone for 6 dpf *scxa*$^{+/kg170}$ incross embryos, showing as flatmounts of the pharyngeal skeleton (ventral view, left) or neurocranium (dorsal view, right). **B-C.** 13 dpf *scxa*$^{+/kg170}$*;Tg(col2a1:mCherry)* incross juveniles shown for live mCherry expression (ventral view, B) and Alcian blue and Alizarin red double staining (pharyngeal skeleton, ventral view, C, left and neurocranium, dorsal view, C, right). No dramatic differences are detected beyond normal variation.

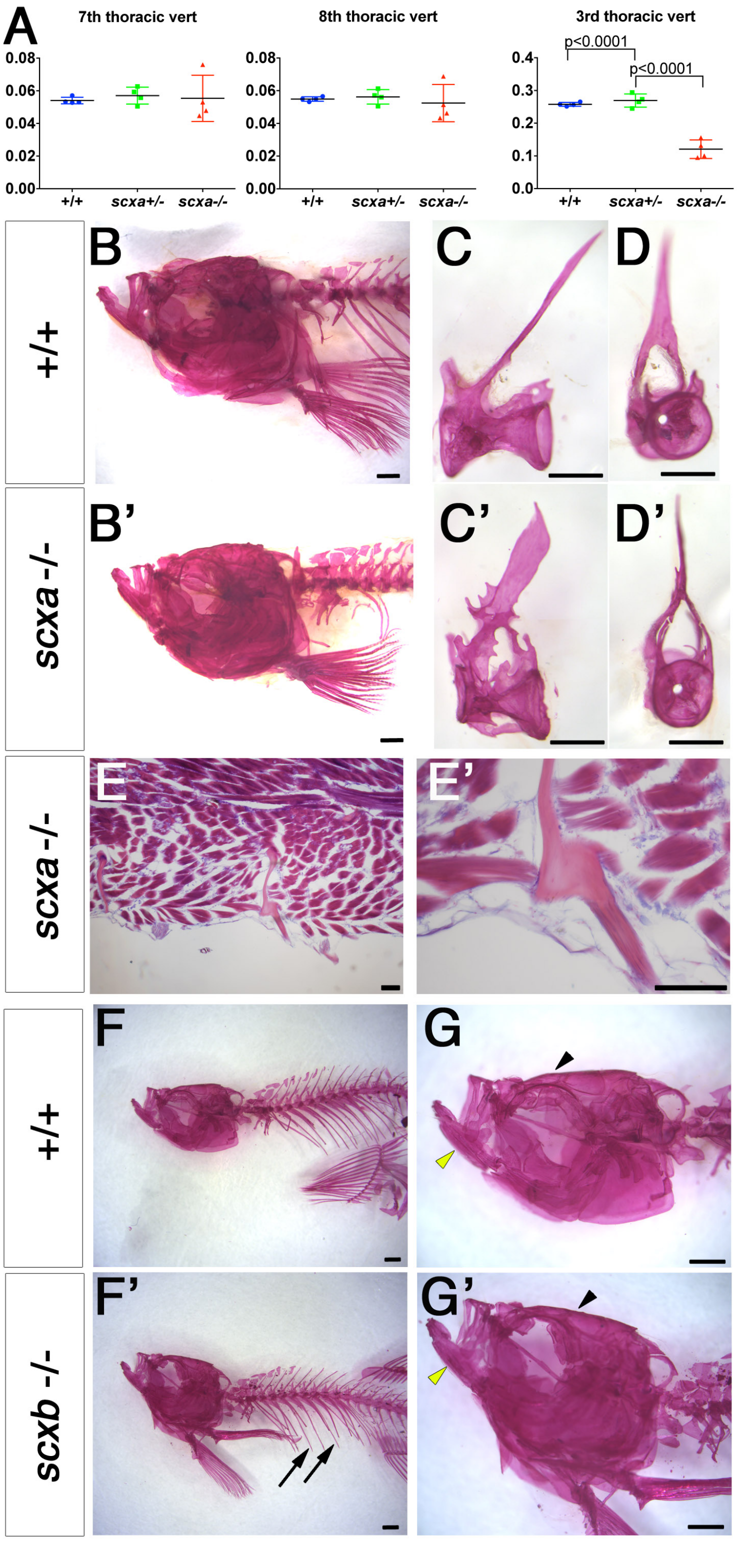

Figure S5. Adult *scxa* mutants show skeletal defects in trunk but not in skull.
A. Vertebral centrum volumes calculated from µCT scans of genotyped adults from a *scxa*$^{+/-}$ incross (n=3 per genotype) using the minimum volume possible around the neural canal, excluding (for 7th and 8th thoracic vertebrae) or including (for the 3rd thoracic vertebra) all processes, trabeculae, spines and ribs in a transverse view. Vertebrae centrum alone is similar between all genotypes, but when the above skeletal elements were added, volume in *scxa*$^{-/-}$ fish is significantly smaller than siblings. One-way ANOVA statistics with Tukey's post-hoc test performed, p-values indicated. **B-D', F-G'.** Alizarin Red staining for adult *scxa*$^{-/-}$ (B'-D') and siblings (B-D) or *scxb*$^{-/-}$ mutants (F',G') and their siblings (F,G). Skull of *scxa* mutants appeared normal whereas staining in ribs was missing**.** Skull (arrowhead), jaw (yellow arrowhead) and ribs (arrows) looked normal in *scxb* mutants. Dissected thoracic 12th vertebrae from *scxa*$^{-/-}$ mutant and sibling are shown in lateral and frontal views for details of bony growth in arches (C',D' compared with C,D). **E, E'**. Adult *scxa* mutant zebrafish sagittal paraffin section stained with Hematoxylin and Eosin and Alcian blue showing fractured and healed rib fragment (magnified in E'). Scale bars, 100µm in E,E', 1mm in B,B',F,F', 0.5mm in C-D'.

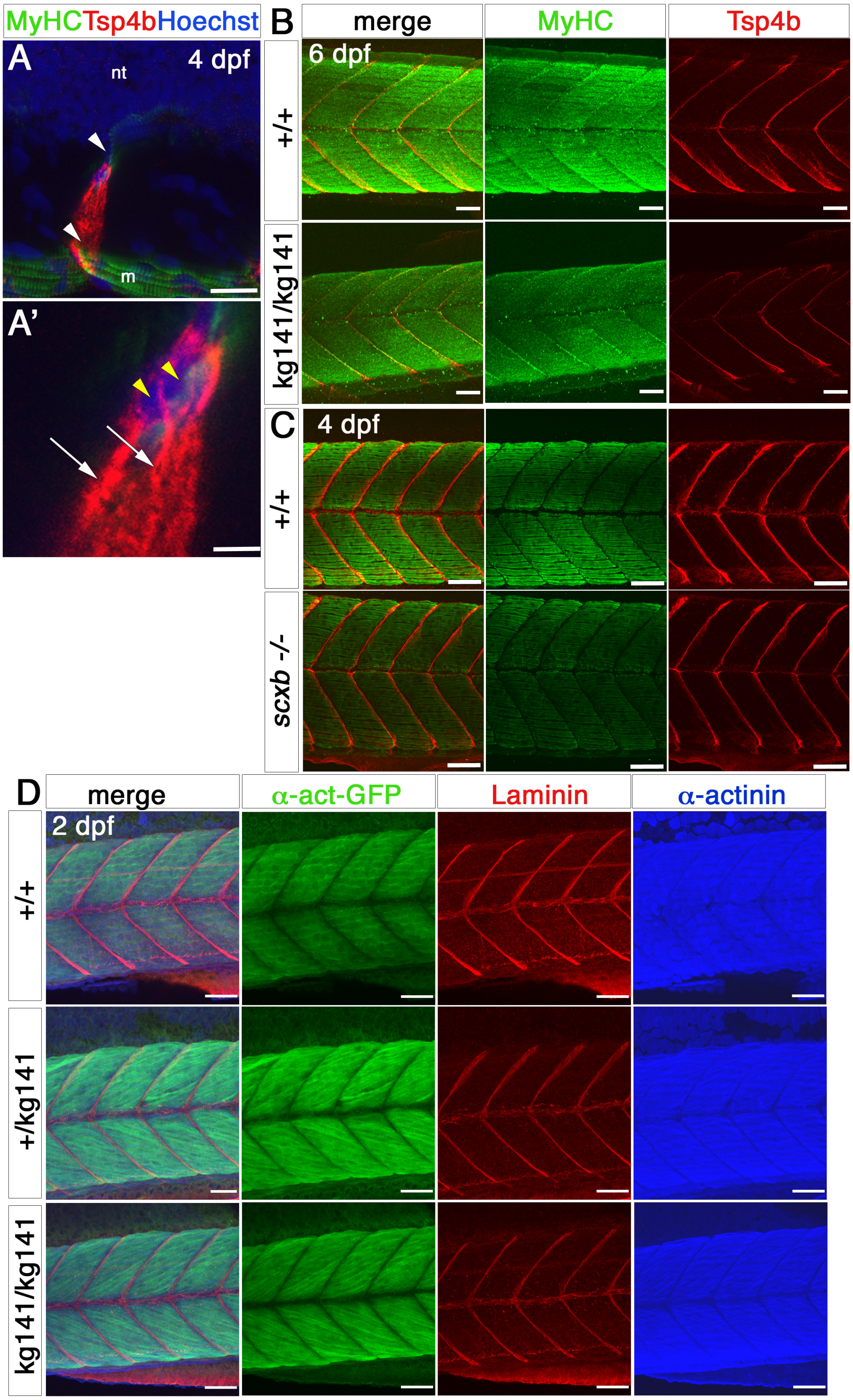

Figure S6. Myotendinous junctions and muscle of *scxa*^{kg141} and *scxb* mutants appear normal. **A-A'.** Small confocal stacks of immunofluorescence for MyHC (A4.1025, green), Tsp4b (red) and nuclear stain (Hoechst) of 4 dpf wt embryos somites 11,12, in lateral view, showing cells (magnified in A') at the edge of myosepta (white arrowheads) surrounded by the somites and the neural tube (nt), in which single nuclei (yellow arrowheads) are surrounded by Tsp4b matrix stain and extend processes of Tsp4b along the myosepta (arrows). m, muscle fibres. **B,C.** Lateral view of somites 11-14 of confocal stacks for immunofluorescence of MyHC (A4.1025, green), Tsp4b (red) of 6 dpf embryos from a *scxa*$^{kg141/+}$ incross (B) or 4 dpf of a *scxb*$^{+/-}$ incross (C). No major differences in either MyHC or Tsp4b are detected. **D.** Lateral view of confocal stacks for GFP, Laminin and sarcomeric alpha-actinin of somites 11-14 from 2 dpf *scxa*$^{kg141/+}$*;Tg(actc1b:egfp)*^{zf13} incross embryos. No major differences between genotypes are detected. All scale bars 50μm, except A-A', 20μm.

Figure S7. ***Video: Swim behavior differences between mutant and sibling adult fish.*** Split screen showing two video fragments shot from above, taken from the original videos of *scxa*$^{+/kg170}$ and *scxa*$^{kg170/kg170}$ adult fish swimming in a tank.

Figure S8. Parameter file for downloading for use in the "Modular image analysis" software.
